# On the Electron-Induced Reactions of (CH_3_)AuP(CH_3_)_3_: A Combined UHV Surface Science and Gas-Phase Study

**DOI:** 10.3390/nano12152727

**Published:** 2022-08-08

**Authors:** Ali Kamali, Elif Bilgilisoy, Alexander Wolfram, Thomas Xaver Gentner, Gerd Ballmann, Sjoerd Harder, Hubertus Marbach, Oddur Ingólfsson

**Affiliations:** 1 Department of Chemistry and Science Institute, University of Iceland, Dunhagi 3, 107 Reykjavik, Iceland; 2 Physikalische Chemie II, Friedrich-Alexander Universität Erlangen-Nürnberg, 91058 Erlangen, Germany; 3 Inorganic and Organometallic Chemistry, Universität Erlangen-Nürnberg, 91058 Erlangen, Germany; 4 Carl Zeiss SMT GmbH, 64380 Roßdorf, Germany

**Keywords:** focused-electron-beam-induced deposition (FEBID), dissociative ionization, ultra-high vacuum, gold deposits, Auger electron spectroscopy (AES), HV gas-phase study, quantum chemical calculation, low-energy electrons, electron-induced mechanism

## Abstract

Focused-electron-beam-induced deposition (FEBID) is a powerful nanopatterning technique where electrons trigger the local dissociation of precursor molecules, leaving a deposit of non-volatile dissociation products. The fabrication of high-purity gold deposits via FEBID has significant potential to expand the scope of this method. For this, gold precursors that are stable under ambient conditions but fragment selectively under electron exposure are essential. Here, we investigated the potential gold precursor (CH_3_)AuP(CH_3_)_3_ using FEBID under ultra-high vacuum (UHV) and spectroscopic characterization of the corresponding metal-containing deposits. For a detailed insight into electron-induced fragmentation, the deposit’s composition was compared with the fragmentation pathways of this compound through dissociative ionization (DI) under single-collision conditions using quantum chemical calculations to aid the interpretation of these data. Further comparison was made with a previous high-vacuum (HV) FEBID study of this precursor. The average loss of about 2 carbon and 0.8 phosphor per incident was found in DI, which agreed well with the carbon content of the UHV FEBID deposits. However, the UHV deposits were found to be as good as free of phosphor, indicating that the trimethyl phosphate is a good leaving group. Differently, the HV FEBID experiments showed significant phosphor content in the deposits.

## 1. Introduction

The need for ever-smaller and precisely manufactured nanostructures in fields such as plasmonics [1], the semiconductor industry [2,3], and in nanoelectronics [4] is one of the drivers in the current development of new and emerging nanofabrication techniques. Focused-electron-beam-induced deposition (FEBID) has high potential in this regard since it is capable of creating nanostructures with precise shapes and position control on basically any substrate. In FEBID, a highly focused electron beam of a scanning electron microscope, in ultra-high vacuum (UHV) or high vacuum (HV), is utilized to induce the fragmentation of adsorbed precursor molecules. The desired structures are built up from non-volatile fragments, while the volatile ones are pumped away [5,6,7].

Controlled localized electron exposure enables the lithographic patterning of practically any shape through direct-write, maskless, and resist-free material deposition. Furthermore, such deposition may be realized on both planar (e.g., Si, SiO_2_) and non-planar substrates (e.g., cantilevers) [7]. FEBID is not only of interest in fundamental research but also in industrial applications [8]. The ability to repair UV and EUV lithography masks [8,9,10], the generation of magnetic nanostructures for magnetic logic circuits [11], and tip fabrication on cantilevers for scanning probe microscopy [7,12,13,14] are examples of industrially relevant applications.

One major challenge in FEBID is that deposits created from organometallic precursors are rarely exclusively composed of the targeted metal (i.e., 100 at.% metal purity). This limits the range of applications for nanostructures created via FEBID [1,15,16]. The purity of the deposition depends strongly on the utilized precursor and the writing parameters such as primary beam energy/current, beam diameter, and replenishment time [17]. It has been shown that it is possible to obtain almost pure iron [18] structures from Fe(CO)_5_ (>95 at.%) or tungsten [19] from WF_6_ (>97 at.%). For gold, however, which has gained high interest, especially in the field of plasmonics [1,20,21], it has been challenging to find a suitable precursor [22,23,24,25]. The most successful gold-based precursors reported in the literature are ClAuCO and ClAuPF_3_ [26,27]. In these studies, it is reported that using these two Au-based precursors, it is possible to obtain pure gold structures on standard SiO_2_ substrates by varying the writing parameters (i.e., primary beam energy, beam current, or dwell time). However, these precursors are difficult to handle due to their pronounced air and water sensitivity and thermal instability. The most investigated precursors for FEBID of gold in the literature are dimethyl gold acetylacetonate [(Me)_2_Au(acac)] [28] and its fluorinated derivatives, dimethyl gold trifluoracetylacetonate [(Me)_2_Au(tfac)] [29,30] and dimethyl gold hexafluoroacetylacetonate [(Me)_2_Au(hfac)] [31]. The corresponding deposits consist of gold cores embedded in a carbonaceous matrix [32]. The co-injection of water as an oxidative enhancer, on the other hand, has been shown to lead to pure gold structures from (Me)_2_Au(tfac) as a precursor [33]. Nonetheless, it would be more convenient to have stable, high-vapor pressure precursors, yielding high-purity deposits in one step, rendering the use of additional purification methods unnecessary.

The electron-induced decomposition mechanisms of FEBID-relevant precursor molecules have been investigated in combined gas-phase and surface science studies [17,34,35,36]. The corresponding results suggest that deposition is mainly initiated by reactions between precursor molecules and low-energy electrons [35,36]. In FEBID, these low-energy electrons are secondary electrons resulting from the interaction of the primary electron beam with the substrate. In general, the electron-induced dissociation of precursor molecules can proceed through four different processes [37]: dissociative electron attachment (DEA), dissociative ionization (DI), neutral dissociation (ND), and dipolar dissociation (DD).

In dissociative ionization [37,38], the focus of the gas-phase section of the current study, electrons with energies equal to or larger than the ionization energy impinge on the parent molecules, leading to their ionization. As a result, the parent ion may undergo dissociation, yielding positively charged and neutral fragments. The DI process can be represented by Equation (1):

(1) $$AB+e^{-}\to AB^{+*}+2e^{-}\to A^{+}+B^{\cdot }+2e^{-}$$

where “*” denotes that the fragments may be in vibrationally and/or electronically excited states. The DI process is a direct, non-resonant scattering process with an interaction time in the order of ~10^−16^ s and starts at or above the ionization energy of a parent molecule (~6–8 eV). The thermochemical threshold for the formation of cations in dissociative ionization can have effects on the extent of fragmentation and, in the simplified case of a diatomic molecule, it can be formulated as:

(2) $$E_{th}\left(A^{+}\right)=BDE\left(AB\right)+IE\left(A\right)$$

where BDE(AB) is the bond dissociation energy of AB, and IE(A) is the ionization energy of the fragment A. For more complex fragmentation processes of polyatomic molecules, where multiple bonds may be broken and new bonds may be formed, the threshold energy $E_{th}$ for the formation of a given fragment is:

(3) $$E_{th}=\sum BDE_{broken}-\sum BDE_{formed}+IE_{\left(fragment\right)}$$

Here, $BDE_{broken}$ and $BDE_{formed}$ are the bond dissociation energies of the bonds broken and the bonds formed in the process, respectively, and $IE_{\left(fragment\right)}$ is the ionization energy of the neutral parent fragment of the cationic fragment observed.

In general, the total dissociation cross-section for a given DI process shows a smooth increase with increasing electron energy and a maximum at around 50–100 eV. At higher energies, the interaction time of the electron with the molecule decreases, and consequently, the total DI cross-section decreases [37]. It has been demonstrated that DI is effective for many FEBID precursors (see, e.g., [39,40,41]) and may thus play a significant role in the deposition process.

The current study investigated the production of Au structures with relatively high metallic content from (CH_3_)AuP(CH_3_)_3_ as a precursor using an SEM setup in UHV. This was carried out in combination with a corresponding gas-phase DI study with the same precursor, yielding important insights into fundamental aspects of the FEBID process. A previous FEBID study on this precursor under HV conditions led to promising results with 19–25 at.% Au from (CH_3_)AuP(CH_3_)_3_ [25]. The use of an ultra-high vacuum (UHV) setup is suitable to reduce unwanted deposits from residual gases and thus reduce contamination effects in the FEBID structures. Therefore, the UHV approach was expected to enable better controlled deposition. This has been demonstrated previously for the precursors Fe(CO)_5_ and Co(CO)_3_NO. Under UHV, these precursors yielded metallic contents higher than 90 at.% and 80 at.%, respectively [18,42]. These values were compared to HV studies which yielded up to 70–85 at.% Fe [7,43] and 40–50 at.% Co [44,45,46] from these precursors, respectively. Therefore, we aimed to test and perform FEBID with this gold precursor, using a UHV-SEM setup with the combination of mass spectrometry (MS) and local Auger electron spectroscopy (AES). To provide a deeper understanding of the underlying electron-induced reaction pathway(s) in the deposition, DI of (CH_3_)AuP(CH_3_)_3_ was also studied in the gas phase under single-collision conditions. Quantum chemical calculations of the threshold energies for selected ion fragments observed in DI were also presented and compared to the respective experimental appearance energies to identify the most probable fragmentation pathways.

## 2. Materials and Methods

***Precursor synthesis.*** Methylgold(I) trimethylphosphine ((CH_3_)AuP(CH_3_)_3_) was synthesized by following the steps as described in the literature [47]. The synthesis was performed under nitrogen atmosphere, using pre-dried solvents and standard Schlenk techniques. The starting material was H[AuCl_4_]·3H_2_O, which was obtained in the form of orange crystals by dissolving gold metal in aqua regia, evaporating all liquids, adding concentrated HCl, and evaporating all liquids again. The quality of the compound was checked and confirmed via ^1^H and ^31^P NMR spectroscopy and via elemental analysis (C, H, and N values showed a maximum deviation of 0.5%).

***Precursor handling.*** The (CH_3_)AuP(CH_3_)_3_ precursor was kept at 253 K in a refrigerator inside a glove box (O_2_ < 0.1 ppm) and later filled into a stainless-steel precursor storage holder at room temperature under nitrogen atmosphere in the glove box. The precursor holder had a small glass window to enable the precursor quality to be checked visually. The filled storage holder was wrapped in aluminum foil to avoid photodecomposition during the experiments and attached to the analysis chamber.

***Deposition.*** FEBID structures were fabricated in a modified commercial UHV system (Scienta Omicron GmbH, Taunusstein, Germany) with a base pressure of *p* < 2 × 10^−10^ mbar. For the mass spectrometry (MS) of the (CH_3_)AuP(CH_3_)_3_ precursor in the gas phase, a quadrupole mass spectrometer (Prisma QMS 200 M, Pfeiffer Vacuum GmbH, Aßlar, Germany ) that was mounted to the UHV chamber was used, and the precursor was sublimed into the chamber at room temperature (298 K). The system included a UHV-compatible scanning electron microscopy (SEM) column (Leo Gemini, nominal resolution better than 3 nm); the latter was also used for FEBID. In addition, a hemispherical electron energy analyzer (EA125, Scienta Omicron GmbH, Taunusstein, Germany) enabled local Auger electron spectroscopy (AES). The electron beam settings during FEBID were a primary electron beam energy of 5 keV and a beam current of 3 nA. The lithographic processes were controlled through custom-developed software based on LabVIEW 8.6 (National Instruments, Austin, TX, USA) and a high-speed DAC PCIe-card (M2i.6021-exp, Spectrum Elektrotechnik GmbH, München, Germany) [48]. The lithographic processes were performed with a step size of 6.2 nm and a sweep number of 100. SEM images were acquired at a beam energy of 15 keV and a current of 400 pA with SmartSEM (Zeiss, Oberkochen, Germany). Minor contrast and brightness adjustments were applied. A commercially available SiO_2_ (230 nm)/Si (111) substrate was used as delivered to create FEBID structures.

The precursor gas was allowed to effuse into the system through a nozzle in close proximity to the sample surface. Based on simulations using GIS Simulator (version 1.5) [49], the local pressure increase at the sample surface was estimated to be a factor of 30, and the chamber pressure was kept at 1.3 × 10^−7^ mbar, which corresponded to a local pressure of ~4.0 × 10^−6^ mbar at the substrate’s surface.

***Gas-Phase Study.*** The DI experiments were carried out with a crossed electron/molecular beam apparatus that has been described in detail elsewhere [50], and only a brief description is given here. Electrons were emitted from a tungsten filament and guided with a stack of electrical lenses through a trochoidal electron monochromator to generate a quasi-monoenergetic electron beam. The full width at half maximum (FWHM) of the electron energy spread was about 140 meV during the experiments. The temperature of the monochromator was kept at 393 K with two halogen lamps to avoid the condensation of precursor molecules or background contaminations on its electronic lens components. In the interaction section of the instrument, the electron beam crossed a molecular beam of (CH_3_)AuP(CH_3_)_3_, generated by sublimation at room temperature (298 K) through an effusion stainless-steel capillary inlet. The background pressure in the chamber was in the order of 2–3 × 10^−8^ mbar, and the working pressure was in the range of 7–9 × 10^−7^ mbar. Ionic fragments formed, as a result of the collision of the electrons with the precursor molecules, were extracted into a quadrupole mass spectrometer (EPIC 1000, Hiden Analytical Warrington, UK) and analyzed and detected with a channelton electron multiplier. Mass spectra were recorded at fixed electron energy by scanning through the relevant *m*/*z* range, and ion yield curves were recorded at fixed *m*/*z* by scanning through the relevant electron energy range. The positive ion yields were normalized relative to the cross-section of the formation of Ar^+^ from Ar at 50 eV recorded after all fragment ion measurements [51]. The appearance energies for positive ion fragments were evaluated by fitting a Wannier-type model function to the onset region of the respective ion yields, as has been described in detail [52], and the energy scale was calibrated with reference to the first ionization energy of Ar [51]. 

***Quantum chemical calculations.*** The current calculations were carried out with the ab initio quantum chemistry program package ORCA, version 4.1 [53]. Geometry optimizations were carried out at the density functional level of theory (DFT) using the meta-generalized gradient approximation (meta-GGA) TPSS functional and the valence triple-zeta polarization basis set def2-TZVP. The D3(BJ) dispersion correction by Grimme et al. was included in the calculations [54]. For closed-shell systems, the restricted Kohn–Sham (RKS) formalism was used, and the unrestricted Kohn–Sham (UKS) formalism was used for open-shell systems. The geometry optimizations were conducted with tight SCF settings, and the single-point energies, at the TPSS/def2-TZVP level of theory, were calculations with normal SCF settings. The TPSS/def2-TZVP approach was chosen based on studies conducted by Kepp [55] and Goel et al. [56], where they found the TPSS/def2-TZVP level of theory to give more reliable bond energies and structures of gold clusters compared to other functionals such as B3LYP, M06, and PBE0. Positive values of harmonic vibrational frequencies were confirmed at the same level of theory and were used to derive zero-point energies and thermal energy corrections at 298 K for the neutral parent molecule and all fragments. Additionally, single-point energies of the optimized geometries were calculated at the coupled cluster level of theory. The coupled cluster calculations were performed using domain-based local pair natural orbitals with single, double, and perturbative triple excitations, DLPNO-CCSD(T) [57,58,59]. These calculations were carried out with normal PNO settings and the valence quadruple-zeta basis set QZVPP, with two sets of polarization functions.

The threshold energy for each fragment was calculated from the single-point energies of the relaxed structures, both at the DFT and DLPNO-CCSD(T) level of theory. This was conducted by subtracting the total energy of all fragments formed in the respective processes from the total energy of the parent molecule. The respective ZPVEs and thermal energy corrections were included in all cases.

## 3. Results and Discussion

### 3.1. UHV-FEBID Study

#### 3.1.1. Promotion of the Intact Precursor into the Gas Phase and Its Stability

**Figure 1:**
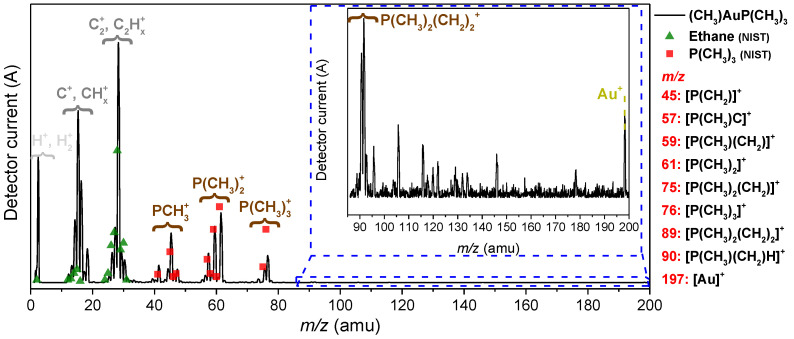
Mass spectrum of (CH_3_)AuP(CH_3_)_3_, recorded at room temperature and a precursor pressure of 1.3 × 10^−7^ mbar (black line). The reference spectrum for trimethylphosphine (P(CH_3_)_3_) from the NIST database is shown as red squares, and that for ethane as green triangles. A 60× magnified region from *m*/*z* 85 to 200 is shown in the inset within the blue dashed lines. The observed precursor-related fragments, along with their *m*/*z*, are listed at the right-side of the graph.

To characterize the precursor prior to the FEBID experiment, (CH_3_)AuP(CH_3_)_3_ was dosed into the UHV chamber via the gas-injection system (GIS) and monitored with a mass spectrometer. Figure 1 depicts a positive ion mass spectrum recorded under FEBID conditions, i.e., at a chamber background pressure of 1.3 × 10^−7^ mbar. Below *m*/*z* 40, the mass spectrum exhibited contributions of CH*_n_* and C_2_H*_n_* with an admix of nitrogen- and oxygen-containing fragments. The significant contribution at and around *m*/*z* 28 may in part have been derived from ethane as a decomposition product, and for comparison, the relative intensities from the NIST DI spectra of ethane are shown as green triangles in Figure 1. The relative intensities around *m*/*z* 28 agreed well with the respective DI products from ethane, considering that *m*/*z* 28 may have drawn additional intensity from nitrogen originating from the precursor filling process (see the Experimental Section).

This was consistent with the proposed ethane formation in the thermal decomposition of (CH_3_)AuP(CH_3_)_3_ [60] and upon its decomposition on active surfaces. However, as the CH*_n_*^+^ intensities around *m*/*z* 15 were considerably higher than what was to be expected from DI of ethane, we considered these contributions to stem mainly from direct CH*_n_*^+^ loss from the precursor. Furthermore, both the contributions around *m*/*z* 28 and *m*/*z* 15 most likely contained admixtures of residual gases. In good agreement with the NIST electron impact mass spectra of trimethylphosphine [61] (Figure 1, red-colored squares), clear contributions from the trimethylphosphine group were present in the mass spectrum. These were at *m*/*z* 45 (PCH_2_^+^), *m*/*z* 57, 59, and 61 (reflecting P(CH_3_)_2_ along with the loss of one and two hydrogens) as well as at *m*/*z* 75 and 76 (reflecting P(CH_3_)_3_ along with the loss of one hydrogen). Additionally, Au^+^ (*m*/*z* 197) was clearly observable in the mass spectrum, indicating the promotion of the intact precursor into the gas phase at room temperature. This was confirmed in the gas-phase experiments discussed in the DI section. With reference to *m*/*z* 61, the DI peak ratios for the *m*/*z* ratios of 61, 75, 76, and 197 (Au^+^) reported in the literature [47] were 100:33:74:1. The corresponding relative intensity reported for the parent ion ([(CH_3_)AuP(CH_3_)_3_]^+^, *m*/*z* 288) and the parent after the loss of one methyl group ([AuP(CH_3_)_3_]^+^, *m*/*z* 273) were found to be about 30 and 200, respectively. This was a clear sign of a significant presence of the intact precursor in the gas phase in this experiment. Similarly, from the peak heights of the mass spectrum shown in Figure 1, we derived the relative intensity ratios 100:20:39:0.4 for these fragments (*m*/*z* 61:75:76:197). The literature data cited here were recorded with a sector field mass spectrometer, while the current mass spectra from the UHV chamber were recorded with a quadrupole with the higher *m*/*z* limit 200; notably, the transmission properties of these instruments were very different. Further, Au^+^, *m*/*z* 197, was at the *m*/*z* limit of this quadrupole, where the transmission efficiency was comparatively low and the parent cation, [(CH_3_)AuP(CH_3_)_3_]^+^, *m*/*z* 288 was outside its *m*/*z* range. Considering this, the relative intensities of the *m*/*z* ratios observed were in good agreement. The relatively high contribution of the parent ion and the parent ion after the loss of one methyl group in the sector field experiments [47] confirmed the delivery of the intact precursor molecule into the gas phase in this experiment. From the comparison of the intensity ratios, we anticipated that that was also the case in the current experiment. This was further supported by the deposition and gas-phase DI experiments discussed below. With respect to volatility and stability, (CH_3_)AuP(CH_3_)_3_ at room temperature was found to be sufficiently volatile to sublime and establish a chamber background pressure of 1.3 × 10^−7^ mbar (equivalent to ~4.0 × 10^−6^ mbar at the surface; see Experimental Section). Compared to earlier FEBID studies with this setup [18,42], using other precursors, this chamber pressure was low (1.3 × 10^−7^ mbar, this study, vs. 3.0 × 10^−7^ mbar, earlier FEBID studies). However, as is discussed in the deposition section, it was found to be sufficient to create the respective deposits. No change in color or in other aspects of appearance of the precursor was visible in the container of the GIS after one week of operation at room temperature, though the gold content of the deposits was found to drop already after three days (Figure S1a,e). The gold content of the deposits could be re-established by increasing the sublimation temperature to 313 K while maintaining the same chamber pressure of 1.3 × 10^−7^ mbar. However, a further increase in the sublimation temperature to 323 K resulted in a significant drop in the gold content of the deposits, and degradation was visible upon inspection after these experiments. Moreover, directly after the heating process, the precursor color was changed from shiny-white to pink/black, which we attributed to the thermal decomposition of the precursor (Figure S1b,c). The autocatalytic decomposition of (CH_3_)AuP(CH_3_)_3_ on active metal surfaces such as copper has been reported to take place already at room temperature [60] and may have played a role in the current setup, where copper sealings were used in the GIS. In fact, the contact surface of these copper sealings was found to be clearly discolored and darkened when the degradation of the precursor was observed (Figure S1d). It is thus clear that this precursor is sufficiently volatile for FEBID, but the temperature window is narrow, and potentially, the material composition of the gas inlet system plays a role in the decomposition process. Further ex situ stability testing and vacuum thermogravimetry would be valuable in establishing these parameters.

#### 3.1.2. FEBID

**Figure 2:**
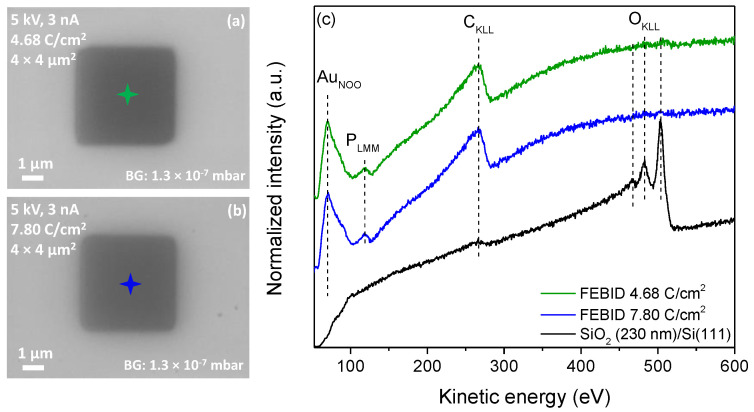
(**a**) SEM image of a 4 × 4 µm^2^ FEBID structure deposited on SiO_2_ from (CH_3_)AuP(CH_3_)_3_ with an electron dose of 4.68 C/cm^2^ and (**b**) with an electron dose of 7.80 C/cm^2^. In both cases, the electron beam parameters are 5 keV and 3 nA, and (**c**) AES of the SiO_2_ substrate prior to deposition (black line) and AES from FEBID structures (green and blue lines). The colored stars in (**a**,**b**) indicate the position where the spectra were acquired.

In the first step, focused-electron-beam-induced deposition experiments were performed on a commercially available SiO_2_ (230 nm)/Si (111) substrate kept at room temperature. Banaszak et al. [62] reported that a thin (40 Å) silicon oxide surface on Si (111) leads to the spontaneous deposition of gold on surface defect sites at 298 K with (CH_3_)AuP(CH_3_)_3_ as a precursor in chemical vapor deposition (CVD) experiments in UHV. In the same study, however, the authors demonstrated that a thicker silicon dioxide film of 5000 Å is not reactive towards the decomposition of (CH_3_)AuP(CH_3_)_3_ at room temperature. It can thus be concluded that a silicon surface with a too-thin silicon oxide layer can be reactive towards the (CH_3_)AuP(CH_3_)_3_ precursor, while thicker layers are inert. Accordingly, the wafer used in the study at hand was selected with a 230 nm silicon oxide layer, which we expected to be inert with respect to the surface-promoted decomposition of the precursor at room temperature. Aligned with the pre-testing, the (CH_3_)AuP(CH_3_)_3_ precursor was sublimed into the chamber from the sample container at room temperature. The corresponding precursor dosage was adjusted for deposition such that a chamber pressure of 1.3 × 10^−7^ mbar was set (local pressure at the sample: ~4.0 × 10^−6^ mbar; see Experimental Section). The chamber pressure was about half the pressure compared to previous FEBID studies performed in the same UHV setup [18,42,63,64]. The relatively low pressure was due to the low volatility of the precursor compared to the well-studied Fe(CO)_5_ and Co(CO)_3_NO precursors [18,42], and correspondingly, a lower deposition rate was expected. It is worth mentioning that due to the thermal instability of the compound, discussed in the previous section, no external heating was applied to the precursor container to increase the vapor pressure. To compensate for the low precursor pressure, a relatively low SEM acceleration voltage of 5 keV was selected to increase the secondary electron yield for the deposition of (CH_3_)AuP(CH_3_)_3_. Using this acceleration voltage, 4 × 4 µm^2^ squares were written using a comparably high beam current of 3 nA. The fabricated FEBID structures were examined with SEM and AES. Figure 2a,b depict SEM images of the FEBID deposits fabricated with electron area exposures of 4.68 and 7.80 C/cm^2^, respectively. Auger spectra were acquired with an acceleration voltage of 15 kV and a beam current of 3 nA. The centers of these rectangles, where the AES spectra were recorded, are marked by green- and blue-colored stars in the corresponding SEM images (Figure 2a and 2b, respectively), and the AES spectra acquired of the bare substrate and the deposited structures are shown in Figure 2c. From the pristine SiO_2_ surface as a reference (black spectrum), only two main AES elements are visible, oxygen and carbon. The low-intensity peak at 272 eV was attributed to C_KLL_ Auger transitions of carbon [65], and the dominating peaks at 468, 483, and 503 eV were assigned to O_KLL_ Auger transitions of SiO_2_ [66]. After deposition with a 4.68 C/cm^2^ electron dose, the O_KLL_ Auger transitions of SiO_2_ vanished, and AES signals appeared at kinetic energies of 69, 120, and 265 eV. These were assigned to Au_NOO_, P_LMM_, and C_KLL_ Auger transitions [66], respectively (Figure 2c, green and blue spectra).

The elemental compositions of the FEBID structures were calculated according to the relative sensitivity factors (*S*) of characteristic AES peaks for each element. To obtain the elemental composition from AES, the following equation was used:

(4) $$C_{x}=\frac{I_{x}}{S_{x}d_{x}}/\sum_{i}\left(\frac{I_{i}}{S_{i}d_{i}}\right)\;$$

where *C* is the atomic concentration, *I* is the integrated spectral intensity, *d* is a scaling factor, and *S* is the relative sensitivity factor [66]. The subscript *x* denotes all values corresponding to the investigated peak, whereas the subscript *i* “counts” through every peak in the spectrum. In the work at hand, peak areas were only compared within one spectrum. As a result, the scaling factor *d*, which was introduced to compensate for errors caused by the different intensity in two different Auger spectra, stayed constant for every peak and could be neglected. The integrated spectral peaks after linear baseline correction of the spectra were used for quantitative evaluation. The atomic concentrations for the FEBID structures shown in Figure 2 were calculated using Equation (4) and the relative sensitivity factors (*S*) [67]; 0.21 for Au_NOO_ at 69 eV, 0.30 for P_LMM_ at 120 eV, and 0.08 for C_KLL_ at 272 eV. These were found to be 31 at.% Au, 67 at.% C, and 2 at.% P (green spectrum), and 34 at.% Au, 65 at.% C and 1 at.% P (blue spectrum).

In previous FEBID studies of the same precursor, CH_3_AuP(CH_3_)_3_, in HV [22,25], the deposit’s composition was determined via EDX spectroscopy and found to be 19–25 at.% Au, 54–62 at.% C, 12–16 at.% P, and 2−7 at.% O. The underlying reaction path determining this composition was suggested to be the removal of one single methyl ligand. Clearly, more significant gold content was observed in the UHV deposits compared to those made in HV. However, in the structures deposited from the CH_3_AuP(CH_3_)_3_ compound in UHV, unexpectedly, more significant carbon content and significantly lower phosphorus content were also observed. However, we note that caution should be taken when comparing the composition of the deposits in the HV study and the current UHV, as EDX is much less surface-sensitive than the here-applied AES. With this information, one might also speculate that the distribution of carbon in the UHV-FEBID structure is not even within the deposit but is enhanced in the surface region.

From the results of this study, however, the very low phosphorus content indicated the efficient removal of the trimethylphosphine ligand during the deposition, while the predominant removal of a single methyl group was more consistent with the HV-FEBID results. In fact, judging from the close to 1:2 Au:C composition of the current deposits, a dimethylphosphine group was dissociated from the precursor and pumped away from the chamber. This may have proceeded through the further decomposition and co-deposition of carbon from dissociating trimethylphosphine ligands or in a concerted electron-induced rearrangement reaction such as:

(5) $$\left({\mathrm{CH}}_{3}\right)\mathrm{AuP}{\left({\mathrm{CH}}_{3}\right)}_{3}\;\overset{e^{-}}{\to }\;\mathrm{Au}{\left({\mathrm{CH}}_{3}\right)}_{2}+P{\left({\mathrm{CH}}_{3}\right)}_{2}↑$$

where Au(CH_3_)_2_ is the deposited species, and P(CH_3_)_2_ is the volatile part that is pumped away (note that the charge location is not considered here).

In FEBID, the deposit’s composition results from the interaction of the precursor molecules with the primary electrons, back scattered electrons, and secondary electrons [7,34,68,69]. Hence, in FEBID experiments, the precursor molecules are subject to interaction with a broad energy range of electrons, from close to 0 eV up to the energy of the primary electrons. Thus, little information on individual fragmentation pathways can be gained from these experiments alone. To further explore these findings, we conducted a comprehensive gas-phase study in which we determined the average carbon and phosphor loss per DI incident and compared these with the current deposit compositions and those in the earlier HV FEBID study by Dorp et al. [25]. In addition, we determined the appearance energies for the dominating fragments and compared these with threshold calculations to identify the most probable processes behind individual fragment formation.

### 3.2. HV Gas-Phase Study

#### Dissociative Ionization in the Gas Phase

**Figure 3:**
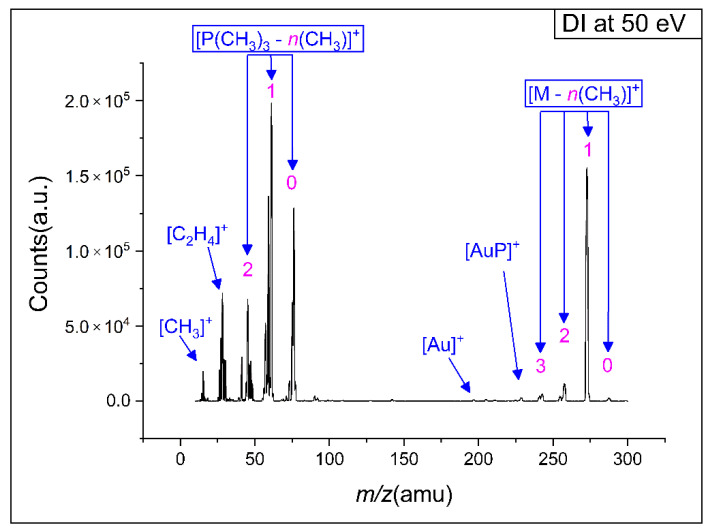
Positive ion mass spectrum of dissociative ionization to (CH_3_)AuP(CH_3_)_3_ precursor at 50 eV incident electron energy.

Figure 3 shows a positive ion, electron impact mass spectrum of (CH_3_)AuP(CH_3_)_3_ in the *m*/*z* range from about 10 to 300. The spectrum was recorded at a 7 × 10^−7^ mbar target gas pressure and 50 eV incident electron energy. The mass spectrum was characterized by two progressions. The first was that of the decomposition of the trimethylphosphine ligand, with the most significant contributions at the *m*/*z* ratios of 45 (PCH_2_^+^), 59 P(CH_3_)CH_2_^+^, 61 P(CH_3_)_2_^+^, 75 (P(CH_3_)_2_CH_2_^+^), and 76 (HP(CH_3_)_2_CH_2_^+^). The second progression was that of CH_3_ loss from the parent molecule, reflected in the *m*/*z* ratios of 273, 258, 243, and 228. These were assigned to the loss of methyl ligand(s) resulting in the formation of [AuP(CH_3_)_3_]^+^, [AuP(CH_3_)_2_]^+^, [AuP(CH_3_)]^+^, and [AuP]^+^. Hence, the two main DI reaction pathways were methyl ligand(s) loss with charge retention on the gold holding fragment, dominated by the loss of a single methyl ligand (*m*/*z* 273), and the dissociation of the charge retaining trimethylphosphine ligand from the gold and its further fragmentation through methyl and hydrogen loss.

As discussed in the study by Marashdeh et al. [22], the (CH_3_)AuP(CH_3_)_3_ precursor is a good candidate for FEBID and CVD due to its comparably good stability at room temperature and volatility under high vacuum. These characteristics stem from the asymmetric crystal structure of (CH_3_)AuP(CH_3_)_3_, which consists of six molecules in a unit cell, in which four molecules have strong aurophilic interactions, while the remaining two molecules are monomeric [22]. Consequently, some of the loosely bound molecules desorb from the crystal under high vacuum. The crystal structure degrades in this process and the now “freed” molecules can go into the gas phase [22,25]. This was reflected in the current gas-phase experiments, in which the intact precursor molecules were readily transported to the chamber at room temperature, as was clear from the significant contribution of the molecular ion after the loss of one methyl group (*m*/*z* 273).

**Figure 4:**
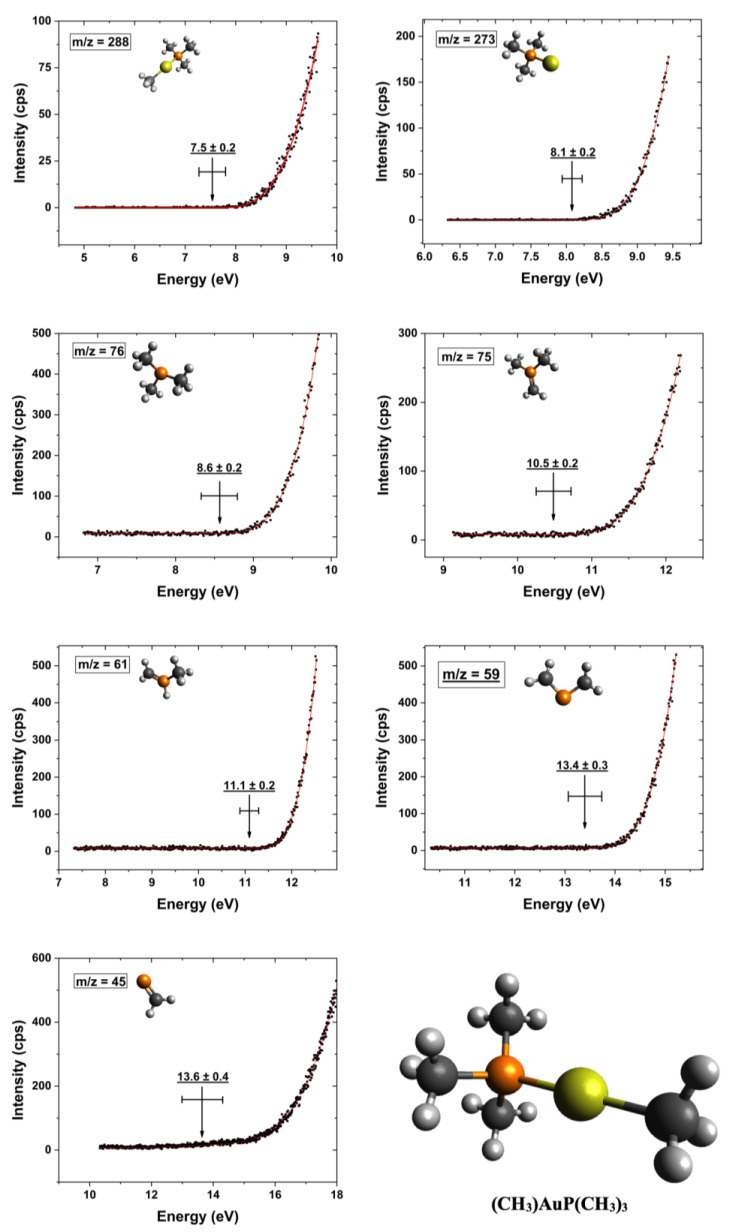
Representative fits to the onset region of the DI ion yield curves for the parent cation and the most dominant positively charged fragments from the (CH_3_)Au(CH_3_)_3_ precursor. The respective Wannier-type fits, appearance energies, and their confidence limits for each ion yield curve are shown, and the respective chemical structure of the intact parent molecule is shown in the right corner.

Figure 4 shows the onset region of the ion yield curves of the most pronounced cations observed in DI of (CH_3_)AuP(CH_3_)_3_. The onset region is shown for the ion yield curves of the intact cation at *m*/*z* 288 and the loss of one methyl group at *m*/*z* 273 ([AuP(CH_3_)_3_]^+^) as well as the P(CH_3_)_3_ rooted fragments: *m*/*z* 76 ([P(CH_3_)_3_]^+^), 75 ([P(CH_3_)_2_(CH_2_)]^+^), 61 ([HP(CH_3_)(CH_2_)]^+^), 59 ([P(CH_2_)_2_]^+^), and 45 ([PCH_2_]^+^). The optimized ionic structures are also shown in the respective graphs. Further, the appearance energies (AEs) and their confidence limits are shown in the individual graphs, along with the respective Wannier-type fits used to determine these energies. The AEs are determined from the average of 3–4 independent measurements and the confidence limits reported are the standard deviations of the mean, rounded to the next 100 meV. In Table 1, the appearance energies are compared to the thermally corrected threshold energies for the respective processes calculated at the TPSS/def2-TZVP and DLPNO-CCSD(T)/QZVPP levels of theory.

For the appearance energy of the molecular cation, i.e., the ionization energy of (CH_3_)AuP(CH_3_)_3_, we determined an experimental value of 7.5 ± 0.2 eV, in good agreement with the threshold values of 7.45 and 7.58 eV, calculated at the TPSS/def2-TZVP and DLPNO-CCSD(T)/QZVPP levels of theory, respectively. Considering the relative intensities in the mass spectrum in the *m*/*z* range of 197 to 288, it was clear that the loss of a single methyl group was the dominating fragmentation pathway leading to the observation of positively charged gold-containing fragments.

In principle, this methyl group could be cleaved directly from the gold by rupture of the Au–CH_3_ bond or from the trimethylphosphine ligand, i.e., rupture of one of the P–CH_3_ bonds. We found the experimentally determined AE for this fragment to be 8.1 ± 0.2 eV, in relatively good agreement with the threshold values of 8.59 (TPSS/def2-TZVP) and 8.38 eV (DLPNO-CCSD(T)/QZVPP), for the loss of the methyl group from the gold, i.e., the formation of AuP(CH_3_)_3_^+^. On the other hand, the calculated threshold values for methyl loss from the trimethylphosphine group were 10.20 and 10.44 eV at the same levels of theory, respectively.

For the dominating trimethylphosphine fragments *m*/*z* 76 and 61, we considered a direct cleavage of the (CH_3_)Au–P(CH_3_)_3_ bond, leaving the neutral (CH_3_)Au moiety with charge retention on the phosphor-containing fragment. For *m*/*z* 75, 59, and 45, which constituted additional hydrogen loss from P(CH_3_)_3_^+^, P(CH_3_)_2_^+^, and PCH_3_^+^, respectively, further neutral fragments were considered.

For the direct dissociation and ionization of P(CH_3_)_3_, *m*/*z* 76, we calculated threshold values of 9.38 and 9.17 eV (TPSS/def-TZVP and DLPNO-CCSD(T)/QZVPP, respectively). These were higher than our experimentally determined AE of 8.6 ± 0.2 eV. Considering hydrogen transfer to the phosphor and the formation of [HP(CH_3_)_2_CH_2_]^+^, as suggested by Bodi et al. [70] for the formation of *m*/*z* 61 ([HP(CH_3_)CH_2_]^+^) in DI of trimethylphosphine, raised the respective threshold values to 9.73 and 9.61 eV, respectively. Considering the formation of the methyl radical and Au, rather than AuCH_3_ as the neutral counterpart to this fragment, increased the respective thresholds further by about 2 eV. For additional hydrogen loss from this fragment, i.e., the *m*/*z* ratio 75, we determined an AE of 10.5 ± 0.2 eV in good agreement with the calculated values of 10.64 and 10.68 eV when considering the formation of HAuCH_3_ as the neutral counterpart. Considering the formation of AuCH_3_ and the hydrogen radical or the formation of [HP(CH_2_)_2_CH_3_]^+^ through hydrogen migration within the cation led to threshold values that were significantly higher (about 1 to 2 eV).

As an alternative to direct methyl loss for the formation of the *m*/*z* ratio 61 in DI of trimethylphosphine, Bodi et al. [70] considered the formation of [HP(CH_3_)(CH_2_)]^+^ through hydrogen migration from one of the methyl groups to the phosphor. At both the G3 and CBS-QB3 levels of theory, they showed a stabilization of about 0.43 eV through this process. Further supported by their kinetic analyses and reaction path calculations, they inferred that this was the dominating channel in the loss of one methyl group from P(CH_3_)_3_ upon DI. This is in good agreement with our calculations, in which we found a stabilization of 0.48 and 0.42 eV, through hydrogen migration, at the TPSS/def2-TZVP and DLPNO-CCSD(T)/QZVPP levels of theory, respectively. The experimental AE for *m*/*z* 61 was 11.1 ± 0.2 eV, and considering (CH_3_)_2_Au as the neutral counterpart; this was in agreement with the threshold energies of 11.16 and 11.20 eV calculated for the [HP(CH_3_)(CH_2_)]^+^ formation at the TPSS/def2-TZVP and DLPNO-CCSD(T)/QZVPP levels of theory, respectively. For direct methyl loss, i.e., the formation of [P(CH_3_)_2_]^+^ without hydrogen migration to the phosphor, we found the respective threshold values to be 11.64 and 11.62, which was in both cases about 0.2 eV above the confidence limits for the AE of *m*/*z* 61. Additionally, we considered the formation of ethane (C_2_H_6_) and Au as neutral counterparts in this process. This led to threshold values of 11.22 and 10.43 eV at the TPSS/def2-TZVP and DLPNO-CCSD(T)/QZVPP levels of theory, respectively. We attribute this difference between the two approaches to the DFT meta-GGA TPSS functional overestimating the atomic energy of Au, and as the CCSD threshold was considerably lower than the experimental AE, we considered (CH_3_)_2_Au to be the neutral counterpart to *m*/*z* 61 instead. The *m*/*z* ratio 59 constituted an additional loss of two hydrogens from *m*/*z* 61 and may have been associated with the neutral counterparts (CH_3_)_2_Au + H_2_, (CH_3_)AuH + CH_4_ or H_2_Au + C_2_H_4_ and the positive fragment P(CH_2_)_2_^+^. The threshold values for these processes at the DLPNO-CCSD(T)/QZVPP level of theory were 13.45 eV for H_2_ formation, 12.93 eV for CH_4_ formation, and 13.07 eV for C_2_H_6_ formation. While the CH_4_ and C_2_H_6_ formation paths were both slightly below the experimental 13.4 ± 0.3 eV AE, there was good agreement with the formation of H_2_ + (CH_3_)_2_Au as the counterparts to the P(CH_2_)_2_^+^ cation.

Finally, the *m*/*z* ratio 45, i.e., PCH_2_^+^, was observed with appreciable intensity. There was a significant number of neutral fragment combinations that could be associated with the formation of this fragment. The preferred assignment through comparison with the calculated thresholds was thus not straightforward. The situation was further complicated as *m*/*z* 45 also showed a quasi-linear rise in the threshold already from about 10 eV. We expected this contribution to stem from the background gas from which *m*/*z* 45 is a common DI contribution, and we accounted for this by replacing the baseline (d) in the Wannier-type function with a linear function a + b*x*. This approach was previously practiced by Fiegele et al. [52] in their determinations of DI thresholds of carbon tetrafluoride, trifluoromethane, methane, and propane. Using this approach, we derived an AE of 13.6 ± 0.4 eV for the formation of this fragment. Within the confidence limits, this agreed with the threshold values for the formation of the neutral counterparts (CH_3_)AuH + C_2_H_6_ and (CH_3_)Au(CH_3_) + CH_4_, which were 13.84 and 13.50 eV, respectively, at the TPSS/def2-TZVP level of theory. The threshold values calculated for other possible reactions were all found to be above the confidence limit, as can be seen in Table 1. Similar to the MS recorded in the UHV-FEBID chamber, we also observed appreciable contributions around *m*/*z* 28 and *m*/*z* 15 in the gas phase HV experiments. Due to the admix of contributions from residual background gases and the potential manifold of different reaction pathways in these *m*/*z* ranges, we did not determine the AEs of these fragments and refer to their discussion above.

**Figure 5:**
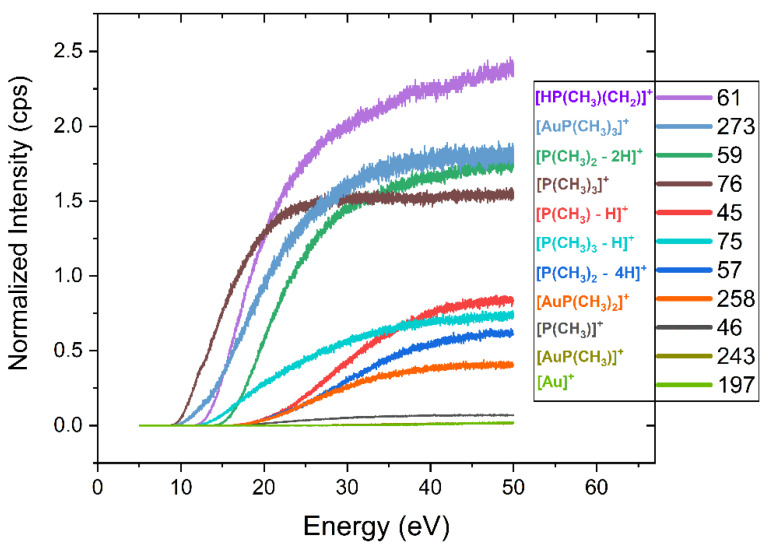
Ion yields for the main positively charged fragments in DI of (CH_3_)AuP(CH_3_)_3_. The ion yields are shown in the incident electron energy range from below the respective thresholds up to 50 eV. All ion yields are normalized with respect to the pressure and the signal intensity of Ar^+^ from Ar at 50 eV incident electron energy.

For better comparison with the deposit composition in FEBID and assessment of the energy dependence of individual reaction channels, Figure 5 shows the ion yield curves for the main fragments observed in the DI mass spectra of (CH_3_)AuP(CH_3_)_3_. These are shown from below the lowest threshold up to about 50 eV and normalized to the pressure and signal intensity of Ar^+^ from Ar at 50 eV incident electron energy.

Table 2 compares the relative contributions of individual fragments integrated over the energy range from threshold to 50 eV with those determined from the peak heights in the mass spectrum shown in Figure 3. In both cases, the intensities reported are normalized to the highest intensity contribution of *m*/*z* 61, set as 100. At the bottom of Table 2, the average carbon and phosphor loss per ionization incident is reported, as well as the respective values from the current UHV-FEBID experiments and the previous HV experiments [25]. For the gas phase, the average carbon and phosphor loss per incident was calculated from the sum of all fragment contributions weighted by the respective carbon and phosphor losses and divided by the total intensity of all DI events. For the [P(CH_n_)_m_]^+^ fragments, the average carbon loss was taken to be what was reflected in the gold-containing neutral counterpart of the respective reactions, shown in bold in Table 1. Other fragments were considered to desorb from the surface. For the deposition experiments, the carbon and phosphor losses were calculated from the difference between the elemental composition of the respective deposits and the stoichiometric ratios of the elements in the intact precursor. Noticeably, in Table 2, the relative integral intensity from the ion yield curves differs from those determined from the peak intensity in the mass spectrum. This is due to the lower integral contribution of the higher threshold fragments as compared to the intensities at 50 eV incident energy. Nevertheless, in both cases, the average carbon loss per ionization incident was about 2, and the average phosphor loss was about 0.8. The Au:P:C composition of the intact parent molecule was 1:1:4, and thus, assuming the desorption of all DI fragments that do not contain gold would result in a deposit ratio of 1:0.2:2 if DI is the dominating fragmentation mechanism.

### 3.3. Dissociative Ionization, UHV, and HV FEBID Composition

With respect to the Au:C ratio, the average carbon loss observed in the gas-phase DI experiments agreed well with the current UHV-FEBID experiments, in which it was also found to be close to 1:2. However, while the phosphor was as good as quantitatively desorbed in the UHV-FEBID experiments, the average phosphor loss per dissociation incident in the gas-phase DI experiments was about 0.8. Hence, in 20% of the DI incidents, the Au-P bond remained intact. This was predominantly due to the stability of the AuP(CH_3_)_3_^+^ ion (*m*/*z* 273) in the gas phase, i.e., loss of the methyl group directly bound to the central gold atom.

**Figure 6:**
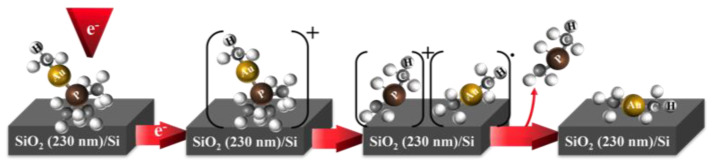
Proposed deposition scheme for the reaction steps of (CH_3_)Au(CH_3_)_3_ in the UHV FEBID. After electron-induced ionization of (CH_3_)AuP(CH_3_)_3_, a positively charged short-lived ion is produced [(CH_3_)Au(CH_3_)_3_]^+^ and fragments to form [P(CH_n_)_2_]^+^ and [(CH_3_)_2_Au]. In the last step, [P(CH_n_)_2_] desorbs from the surface, and [(CH_3_)_2_Au]**^∙^** stays as a deposited fragment.

In the HV-FEBID experiment, the Au:C ratio was determined by EDX to be about 1:2.5 to 1:2.8, and the Au:P ratio was found to be 1:0.63 to 1:0.64. While the slightly higher carbon content of the deposit may have been due to background gas contributions under HV conditions, the significantly higher phosphor content had to be rooted in different decomposition/desorption dynamics in these two experiments [25]. Both experiments were conducted at 5 keV electron energy, and electron current and deposition time did not influence the composition significantly in the HV-FEBID experiments. This difference thus had to be rooted in the different substrates used in the UHV- and HV-FEBID experiments or the difference in background gas partial pressure. The UHV experiments were conducted on a SiO_2_ substrate, a material commonly used for passivation, and it clearly allowed for free desorption of the dissociated phosphor-containing ligands. Moreover, the close to 1:2 Au:C ratio of the deposit indicated that the neutral (CH_3_)_2_Au fragmentation dominated in the electron-induced decomposition of (CH_3_)Au(CH_3_)_3_ at the SiO_2_ surface. This was also the characteristic fragment for the P(CH*_n_*)_2_^+^ loss channels in DI. The AuP(CH_3_)_2_^+^ fragment was, however, also a significant contribution to the total DI ion yield but was not apparent in the electron-induced decomposition at the SiO_2_ surface, as was perceived from the close to quantitative removal of the phosphor. This close to quantitative removal of the phosphor and the 1:2 Au:C ratio in the UHV deposes was consistent with the deposition mechanism, as depicted schematically in Figure 6. In this scheme, electron impact led to a short-lived positive ion that fragmented to form [P(CH*_n_*)_2_]^+^ and [(CH_3_)_2_Au], and while [(CH_3_)_2_Au] stayed on the surface, [P(CH*_n_*)_2_] desorbed.

This mechanism, however, cannot be dominant in the HV FEBID deposition with CH_3_AuP(CH_3_)_3_ on a Si wafer surface. In this experiment, the Au:C:P:O deposit composition was reported to be in the range of 19–25 at.%, Au, 54–62 at.% C, 12–16 at.% P, and 2–7 at.% O. The authors point out that, though not conclusive, this composition may be consistent with a predominant single methyl loss from CH_3_–Au–P(CH_3_)_3_, i.e., loss of the (CH_3_)–Au methyl group [25]. This is in strong contradiction with the UHV FEBID experiments. We cannot offer a conclusive explanation of this marked difference between deposit compositions in the UHV and HV experiments. However, such a significant difference in FEBID composition is not uncommon when comparing deposition under HV and UHV conditions, even on identical substrates [18,42,43,44,45,71]. Most noticeably, in a recent comparative study on FEBID of Pt(CO)_2_Br_2_ and Pt(CO)_2_Cl_2_ [71], as good as quantitative desorption of the halogen was observed under HV conditions while the Pt:Cl and Pt:Br composition of the deposit under UHV conditions remained close to 1:1.56 and 1:1.65, respectively. This has been attributed to reactions with surface water always present in the HV experiments and has been discussed in analogy to the electron-induced decomposition of Pt(NH_3_)_2_Cl_2_, where effective Cl removal through intramolecular reductive HCl formation is observed. Similarly, reductive HCl formation is achieved from surface-adsorbed (η^3^-C_3_H_5_)Ru(CO)_3_Cl [72,73], through in situ exposure to ammonia during electron irradiation. Post and in situ, the oxidative purification of deposits by electron irradiation in the presence of water was also proven efficient in a number of cases, and under HV conditions, a 75% increase in the gold purity of deposits from dimethylgold (III) trifluoroacetylacetonate was attained, reaching 91 at.% Au through oxidative carbon removal in the presence of water [33].

In the HV-FEBID experiment on the present precursor, van Dorp et al. [25] suggested that the presence of O_2_ in the chamber might be responsible for the formation of OP(CH_3_)_3_ fragments. Similarly, it may be speculated that non-volatile OP(CH_3_)_3_ is formed in electron-induced reactions of (CH_3_)AuP(CH_3_)_3_ in the presence of water. This might in part explain the higher amount of phosphor in their experiment as compared to the UHV experiment, but as the oxygen content of the deposits is significantly lower than the phosphor content, this alone cannot account for the observed difference. Notwithstanding the reason for the very different deposit compositions in these experiments, the current UHV study rather supports the conclusion that P(CH_3_)_3_ is a suitable Au(I) ligand in FEBID precursors, while the HV study points towards the contrary.

## 4. Conclusions

In the current study, the suitability of (CH_3_)AuP(CH_3_)_3_ as a precursor for gold deposition in FEBID was explored under UHV conditions, and gas-phase DI experiments and quantum chemical calculations were performed to aid the interpretation of the underlying electron-induced reactions. This potential precursor was found to have sufficient volatility and sufficient stability to be practical as an Au precursor in FEBID, and under the current UHV conditions, 31–34 at.% Au content was achieved at 5 keV electron energy. The Au:C compositing of the deposits was close to 1:2 and in good agreement with the average carbon loss per incident observed in the gas-phase DI experiments, where a significant contribution of the neutral counterparts was found to be (CH_3_)_2_Au. The phosphor, on the other hand, was found to be as good as quantitatively removed in the UHV FEBID experiments, while the average phosphor loss per DI incident in the gas phase was found to be about 0.8. The remaining 0.2 average phosphor per DI incident in the gas, however, could at large be attributed to the loss of the CH_3_–Au methyl group and the formation of AuP(CH_3_)_2_^+^. This reaction channel was clearly not active in the UHV FEBID of (CH_3_)AuP(CH_3_)_3_ on SiO_2_.

While the Au:C:P deposit composition in the current UHV FEBID was found to be about 1:2:0, with as good as quantitative removal of the phosphor, a previous HV FEBID study reported an approximate Au:C:P:O composition of 1:2.6:0.6:0.2. The conclusions of these studies contrast with respect to the suitability of trimethylphosphine as Au(I) ligand in FEBID. Potentially, a part of the phosphor content in the HV experiment may be explained through the electron-induced oxidative formation of trimethylphosphine oxide in reactions with surface water or through reactions with residual oxygen in the chamber. However, other significant factors must also play a role. Compositions of deposits formed under UHV and HV are often significantly different, and like in the current case, the reason(s) for these differences are not obvious. Using a systematic comparison of UHV and HV deposition experiments in deciphering the root(s) of these differences may offer a valuable approach to tailoring better-suited precursors and gaining better control of the compositions of the deposits. From a more general perspective, the approach to combine different methods, namely UHV-FEBID, gas-phase DI experiments, and calculations on a quantum mechanical level proved to be powerful and yielded detailed insights into the mechanisms of electron-induced precursor dissociation and the resulting deposition process.

## Figures and Tables

**Table 1:** Experimental AEs in DI of (CH_3_)AuP(CH_3_)_3_ compared to the threshold values calculated at the TPSS/def2-TZVP and DLPNO-CCSD(T)/QZVPP levels of theory. The best agreements between experiment and theory and the respective reaction paths are signified in bold.

*m*/*z*	Product	TPSS-TZVP	DLPNO-CCSD-QZVPP	AE (eV)
**288**	**[(CH_3_)AuP(CH_3_)_3_]^+^**	**7.45**	**7.58**	**7.5 ± 0.2**
**273**	**[AuP(CH_3_)_3_]^+^ + (CH_3_)**	**8.59**	**8.38**	**8.1 ± 0.2**
	[(CH_3_)AuP(CH_3_)_2_]^+^ + (CH_3_)	10.20	10.44	
**76**	**[P(CH_3_)_3_]^+^ + (CH_3_)Au**	**9.38**	**9.17**	**8.6 ± 0.2**
	[HP(CH_3_)_2_(CH_2_)]^+^ + (CH_3_)Au	9.73	9.61	
	[P(CH_3_)_3_]^+^ + (CH_3_) + Au	12.04	11.54	
**75**	**[P(CH_3_)_2_(CH_2_)]^+^ + (CH_3_)AuH**	**10.64**	**10.68**	**10.5 ± 0.2**
	[P(CH_3_)_2_(CH_2_)]^+^ + (CH_3_)Au + H	12.12	11.86	
	[HP(CH_3_)(CH_2_)_2_]^+^ + (CH_3_)AuH	12.62	12.90	
**61**	**[HP(CH_3_)(CH_2_)]^+^ + (CH_3_)_2_Au**	**11.16**	**11.20**	**11.1 ± 0.2**
	[HP(CH_3_)(CH_2_)]^+^ + C_2_H_6_ + Au	11.22	10.43	
	[P(CH_3_)_2_]^+^ + (CH_3_)_2_Au	11.64	11.62	
**59**	**[P(CH_2_)_2_]^+^ + (CH_3_)_2_Au + H_2_**	**13.46**	**13.45**	**13.4 ± 0.3**
	[P(CH_2_)_2_]^+^ + (CH_3_)AuH + CH_4_	13.18	12.93	
	[P(CH_2_)_2_]^+^ + H_2_Au + C_2_H_6_	13.56	13.07	
	[HP(CH_2_)CH]^+^ + (CH_3_)_2_Au + H_2_	15.22	15.61	
**45**	[PCH_2_]^+^ + (CH_3_)AuH + 2(CH_3_)	17.50	17.04	**13.6 ± 0.4**
	**[PCH_2_]^+^ + (CH_3_)AuH + C_2_H_6_**	**13.84**	**13.20**	
	[PCH_2_]^+^ + Au(CH_3_) + C_2_H_6_ + H	15.32	14.38	
	**[PCH_2_]^+^ + (CH_3_)Au(CH_3_) + CH_4_**	**13.50**	**13.06**	
	[PCH_2_]^+^ + Au + (CH_3_) + C_2_H_6_ + H	17.98	16.76	
	[HPCH]^+^ + (CH_3_)AuH + C_2_H_6_	16.19	15.97	
	[PCH_2_]^+^ + AuH + (CH_3_) + C_2_H_6_	14.85	13.75	
	[HPCH]^+^ + AuH + (CH_3_) + C_2_H_6_	17.19	16.52	

**Table 2:** Relative peak intensities of (CH_3_)AuP(CH_3_)_3_ fragments at 50 eV electron impact energy observed in the DI mass spectrum (Figure 3) and relative integral intensities from thresholds to 50 eV derived from the ion yield curves shown in Figure 5. The FEBID deposits compositions from the current UHV and the previous HV experiments are shown at the bottom of the table.

Fragment	*m*/*z*	Relative DI Yield (Intensity)		Relative DI Yield (Integration)
[AuP(CH_3_)_3_]^+^	273	78.41		79.07
[AuP(CH_3_)_2_]^+^	258	5.64		13.30
[AuP(CH_3_)]^+^	243	2.36		0.36
[Au]^+^	197	0.51		0.29
[P(CH_3_)_3_]^+^	76	64.78		78.98
[P(CH_3_)_3_ − H]	75	33.12		28.47
[HP(CH_3_)(CH_2_)]^+^	61	100		100
[P(CH_3_)_2_ − 2H]^+^	59	68.8		68
[P(CH_3_)_2_ − 4H]^+^	57	26.19		17.98
[P(CH_3_)]^+^	46	14.1		2.54
[P(CH_3_) − H]^+^	45	33.7		24.7
Avrg. C loss per incident		1.94		2.01
Avrg. P loss per incident		0.80		0.76
UHV deposit composition	31–34 at.% Au	65–67 at.% C	1–2 at.% P	
HV deposit composition	19–25 at.% Au	54–62 at.% C	12–16 at.% P	2–7 at.% O

## Data Availability

Data presented in this study are available on request from the corresponding authors.

## Supplementary Materials

The following supporting information can be downloaded at: https://www.mdpi.com/article/10.3390/nano12152727/s1, Figure S1: (a) Gold content of FEBID deposits on SiO_2_ (230 nm)/Si (111) substrate over the timeline of three consecutive experiment runs. Between experiment-run#1 and -run#2 is a pause of three days, and between run#2 and run#3 is one day. (b) The color of freshly filled (CH_3_)AuP(CH_3_)_3_ precursor, and (c) the color of the precursor at the end of experiment-run#3. (d) The color change on copper sealing after the experiment run#3. (e) AES results obtained from the experiments run#1 and run#2.
