# Supplementary material for: On the Electron-Induced Reactions of (CH3)AuP(CH3)3: A Combined UHV Surface Science and Gas-Phase Study
Source: Nanomaterials (Basel). 2022 Aug 8;12(15):2727. doi: 10.3390/nano12152727 (PMC9370483; doi:10.3390/nano12152727)
Supplement: Supplementary file 1 [file nanomaterials-12-02727-s001.zip › nanomaterials-1817623-supplementary.pdf]

Supporting Information

On the Electron-Induced Reactions of $(CH_3)AuP(CH_3)_3$: A Combined UHV Surface Science and Gas-Phase Study

Ali Kamali [1,†], Elif Bilgilisoy [2,†], Alexander Wolfram [2], Thomas Xaver Gentner [3], Gerd Ballmann [3], Sjoerd Harder [3], Hubertus Marbach [2,4,*] and Oddur Ingólfsson [1,*]

1 Department of Chemistry and Science Institute, University of Iceland, Dunhagi 3, 107 Reykjavik, Iceland
2 Physikalische Chemie II, Friedrich-Alexander Universität Erlangen-Nürnberg, 91058 Erlangen, Germany
3 Inorganic and Organometallic Chemistry, Universität Erlangen-Nürnberg, 91058 Erlangen, Germany
4 Carl Zeiss SMT GmbH, 64380 Roßdorf, Germany
* Correspondence: hubertus.marbach@fau.de (H.M.); odduring@hi.is (O.I.)
† These authors contributed equally to this work.

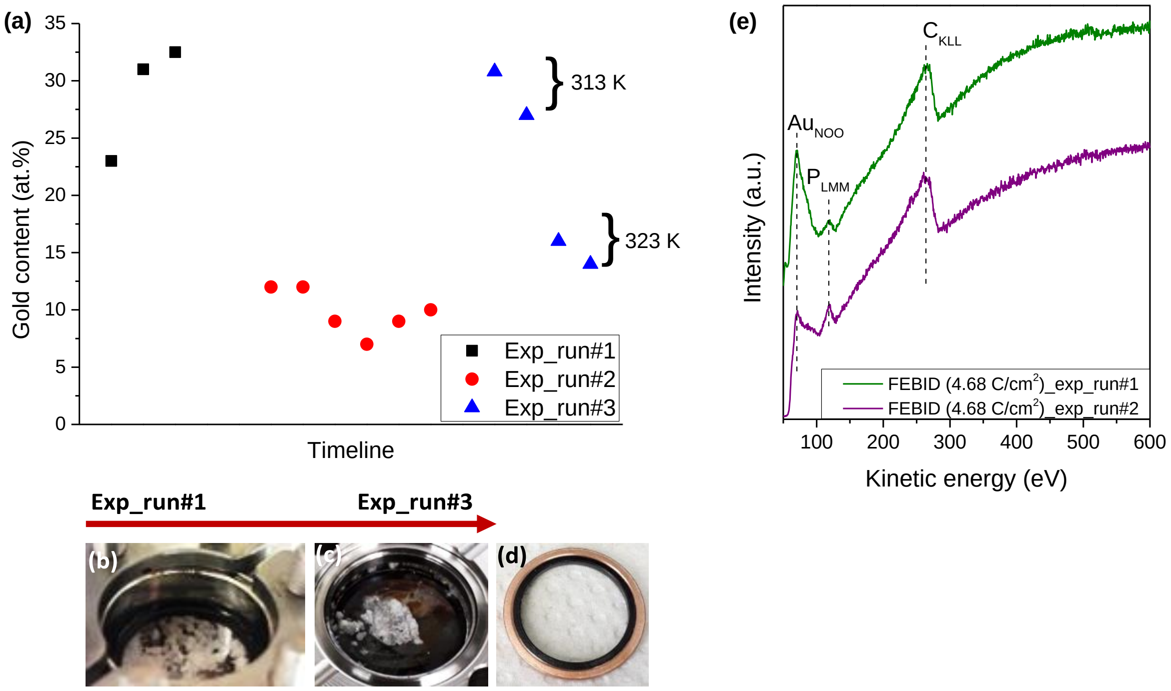

Figure S1. (**a**) Gold content of FEBID deposits on SiO_2 (230 nm)/Si(111) substrate over the timeline of three consecutive experiment runs. Between experiment-run#1 and -run#2 is a pause of three days, and between run#2 and run#3 is one day. (**b**) The color of freshly filled $(CH_3)AuP(CH_3)_3$ precursor, and (**c**) the color of the precursor at the end of experiment-run#3. (**d**) The color change on copper sealing after the experiment run#3. (**e**) AES results obtained from the experiments run#1 and run#2.
